# “Now You See Me, Now You Don’t”: How Digital Consumers Manage Their Online Visibility in Game-Like Conditions

**DOI:** 10.3389/fpsyg.2022.795264

**Published:** 2022-05-13

**Authors:** Rikke Duus, Mike Cooray, Simon Lilley

**Affiliations:** ^1^School of Management, University College London, London, United Kingdom; ^2^Hult International Business School, Ashridge, United Kingdom; ^3^Lincoln International Business School, University of Lincoln, Lincoln, United Kingdom

**Keywords:** visibility, privacy, data, game-like conditions, Propensity to Game (P2G), expression games

## Abstract

Organizations continue to create digital interfaces and infrastructure that are designed to heighten consumers’ online visibility and encourage them to part with their data. The way these digital systems operate and the rules they are governed by are often opaque, leaving consumers to deploy their own strategies for managing their online information sharing with organizations. In this study, we draw upon Erving Goffman’s metaphor of expression games and three forms of concealment or cover moves to explore how consumers, who have been well socialized as digital natives, engage in dynamic and game-like interactions with organizations in an attempt to manage their level of online visibility and information sharing in relation, *inter alia*, to the ‘convenience’ and ‘benefits’ that are afforded to them. Our research is based on in-depth interviews in combination with photo-elicitation with 20 participants. Based on the insight generated, we offer a new framework, ‘Propensity to Game’ (P2G), which present the processual dynamics that characterize these consumers’ evolving and game-like engagements with organizations. These are *Game Awareness*, *Rule Familiarization*, *Player Commitment* and *Game Play.* Our work contributes with new insight into how these consumers actively engage in the orchestration of their online visibility by surfacing the nuanced and multifaceted decision-making and thought processes that they engage in when they, situation-by-situation, decide on the tactics and methods to use in their efforts to manage the data and information they share with organizations.

## Introduction

Since at least the inauguration of market research, organizations have had a keen interest in capturing, accumulating, and analyzing data related to existing and potential customers of their products and services to understand their interests, preferences, and experiences with a view to developing predictive capabilities about future demands and aspirations, effective targeted advertising, new product development and up- and cross-selling of products (Mariani and Wamba, 2020; Ruckenstein and Granroth, 2020). With the gradual but persistent transition of organization-consumer touchpoints to digital platforms and systems, organizations have had near free reign for several decades to design digital infrastructure and systems that, not only, capture consumers’ digital traces, but also encourage them to share an increasing amount of information about themselves (e.g., see Cusumano et al., 2021). Detailed in other work in this area (e.g., Flyverbom, 2016; Bloom, 2019; Zuboff, 2019), all or aspects of our ‘data doubles’ (Ruppert, 2011) that are so produced are of demonstrable value to those able to access, analyze and trade them. In consequence, the resources from which they are made and the rights to their worth are themselves becoming ever more visible sources of contention between the users who leave these traces of identity and the organizations that capture and commercialize them.

Our work contributes to the growing body of research that explores how consumers orchestrate their online visibility and manage their data traces by deploying particular tactics that give them a heightened sense of control and agency in their interactions with organizations. In this paper, we go beyond ascertaining consumers’ attitudes toward privacy in digital spheres by focussing on their behavioral responses, situational decision making and game playing tactics. We capture digitally savvy consumers in the fields of action and surface the nuanced and multifaceted decision-making and thought processes that they engage in when they, situation-by-situation, decide on the tactics and methods to use in their efforts to manage the data and information they share with organizations, which affect their level of online visibility. While our research is informed by the insight generated by others in this field (e.g., Acquisti et al., 2020; Mulligan et al., 2020; Plangger and Montecchi, 2020) we adopt a novel approach by utilizing Goffman’s metaphor of Expression Games and the three forms of concealment or cover moves: covert concealment, minimized sharing and feinting as a lens to explore and surface how consumers knowingly manage their visibilities online as they spend time on digital platforms and ecommerce sites, engage in online shopping, browsing, information gathering and content creation. We undertake this exploration through in-depth interviews in combination with photo-elicitation with experienced users of digital technologies, platforms and devices.

Through this lens of Goffman’s expression games, our research presents a multitude of different situations where there is mutual awareness between the players (i.e., consumers and organizations) and the move made by one player affects the other. In particular, the research draws out the game-like considerations and tactical moves that form part of consumers’ game play. Based on our empirical research, we offer a new framework, ‘Propensity to Game’ (P2G), which conceptualizes our findings and illuminates four states that these consumers typically progress through as part of their game-like interactions with organizations: *Game Awareness*, *Rule Familiarization*, *Player Commitment* and *Game Play.* The P2G framework incorporates two feedback loops which illustrate the dynamic logic of the framework and the continuous work required by consumers to understand new methods and techniques organizations use to heighten their visibility. Therefore, some consumers re-engage with the Game Awareness and Rule Familiarization states to ‘top-up’ their understanding and knowledge of the evolving digital realities. This framework is an important contribution to this field of research as it demonstrates the dynamic nature of visibility management while adding new insight into how, and in which ways, digitally-savvy consumers navigate digital spaces by adjusting their visibility, making calculated and situation-specific acts, and anticipating organizations’ next moves.

## Literature Review

### Contesting the Visibility and Value Nexus in the Digital Economy

We have been concerned with our ability to uphold desired levels of privacy against the interests of the outside world long before our lives became intertwined with digital technologies and platforms. Now, privacy management practices have intensified due to the ability of the digital environment to capture and store even the most subtle of actions (e.g., cursor movements on a webpage).

Flyverbom (2016, p. 7) argues that it is critical to question how information is controlled and understand the processes and mechanisms related to digitalization and datafication that make “certain phenomena and practices visible, and others invisible, in ways that come to guide our attention and contribute to social and political ordering.” It is through addressing these questions that we can come to better understand both “how identities and personal information are curated in digital spaces” (ibid., p. 9) and the increasingly contested value nexus that commercial capture, management, and exploitation of digital data entail (see, for example, Bloom, 2019; Zuboff, 2019). Flyverbom helpfully highlights how these concerns are growing sources of attention for those who seek to understand the affordances (Gibson, 1979) digitalization and datafication bring for platform providers and the commercial associates, along with the concerns for governance and regulation of these new orderings might entail.

Looking back at earlier conceptualizations of ‘privacy,’ Altman (1977) presents privacy as a dynamic process that unfolds as an individual engages in self-other boundary control processes. These processes see the individual enact different behaviors to either open themselves up to others or become more closed off depending on the situation. These dynamic behaviors are enacted to achieve what the individual perceives as being the desired levels of privacy depending on the context and circumstances. As such, it is not a static state of being either open or closed off. Rather, individuals are engaged in privacy seeking behaviors on an on-going basis and in response to the environment they interact with. To take a simple example, players of televised team sports now increasingly put their hands in front of their mouths when talking to team mates on the pitch, doubtless in partial response to prior revelations enabled by a combination of digitally enabled capacities to zoom in on video imagery and the ability to read lips.

In online spheres, consumers are frequently engaged in privacy-seeking behaviors with the aim of curating and personalizing the online environment in a way to only give away the information they are willing to share or trade with organizations. However, this is often not straight-forward for consumers to manage. This is partly due to organizations’ desire to create digital touchpoints that capture consumer data and their disinterest in facilitating mechanisms that empower consumers to actively and transparently manage their levels of privacy and data sharing (Acquisti et al., 2020). There is a sense, however, that the ability for an individual to fully manage their privacy to reach what they consider to be the desired state is often an unattainable aspiration. While Altman’s boundary control process does acknowledge that consumers will switch on and off their visibility depending on the situation and the potential gain from it, in the digital environment most consumers lack critical awareness and digital competences to make decisions that fully reflect their preferences and desires. This is why a desired level of privacy cannot be easily achieved even if it can be determined, leading some consumers to surrender (see Draper and Turow, 2019, for their work on ‘digital resignation’). Moreover, as organizations’ responses are not static, they continue to change their practices to extract information from consumers. So, although consumers learn to adapt, organizations are typically already one step ahead of them.

There are several reasons why consumers often struggle to achieve their desired levels of privacy as explored by Acquisti et al. (2015, 2020). The most common reason is that consumers lack awareness of the methods used by organizations to collect and capture their data (*information asymmetry*). Consequently, organizations have significant opportunities to put in place a multitude of data gathering mechanisms and techniques. In other situations, consumers struggle to comprehend the complexity of the digital environment (*bounded rationality*) and consequently do not read or cannot make practical sense of the privacy policies. Knowing this, an organization can respond by writing long privacy policies that are full of complex legal jargon. A technique often used by organizations to encourage consumers to part with their data is to offer immediate gratification, e.g., access to product discounts, new products, or information in exchange for their data (e.g., requiring the consumer to register/sign up and share information about their preferences). Consumer will often prioritize and value the immediate gain and have less concern for the longer-term implications of profiling and advertising (*present bias*). Ironically, offering detailed privacy settings can encourage greater levels of information disclosure on the part of consumers. Thus, organizations seeking increased openness from their users offer access to amend such settings. A sense of control on the part of the consumer is induced by so doing, which can lead to increased sharing as consumers accept more risk due to their enhanced perceived control (*illusory control*). The last reason we explore here that affects consumers’ ability to achieve their desired levels of privacy and online visibility is the way in which they get used to the risks associated with using digital platforms, especially when these do not change drastically or suddenly (*adaptation*). For organizations, this means that when they change the privacy settings and other related data practices gradually, this gives consumers time to adapt to them and accept the changes.

### Power (Im)balance

When consumers choose to enact behaviors to either open themselves up or become more closed off, their behavior may be triggered by a perceived power imbalance. The power-responsibility equilibrium refers to the unequal power distribution between actors that leads to the ability of an actor to control other actors’ experiences, outcomes and behaviors (Tost, 2015). Those with the power are expected to not misuse their position and “guarantee a trusting market environment” (Bandara et al., 2021). The power-responsibility equilibrium has been applied in the context of data privacy and previous studies (e.g., Dickinson-Delaporte et al., 2020) identify that consumers will take defensive actions, also referred to as ‘power-balancing responses’ when they are concerned that organizations are not actively protecting their privacy. These include deflective behaviors (e.g., use of VPNs, private browsing and identity anonymizers), fabrication of information (e.g., using ‘fake’ information about oneself) and seeking behaviors (e.g., seeking insight from others regarding organizations’ practices) (Bandara et al., 2021). Behaviors like these heighten the need for organizations to “strive toward greater organizational sensitivity around consumer privacy and the current asymmetry in the level of control over personal data” (Puntoni et al., 2021, p. 85).

For years, however, organizations have deployed “dark patterns” in the design of digital interfaces that lead consumers to make choices which, if fully informed and with the presence of alternatives options, they would likely not make (Mulligan et al., 2020). Digital interfaces are often designed to give consumers the illusion of free choice when in fact they are designed to nudge and manipulate them to make *particular* choices, even if they do not match their preceding preferences, and, importantly, ensure that consumers share as much about themselves as possible in the process (Mathur et al., 2019; Acquisti et al., 2020; Waldman, 2020).

For users, there is a lack of transparency in the way digital systems (e.g., apps, ecommerce sites, entertainment platforms, etc.) operate and the rules they are governed by (e.g., Kudina and Verbeek, 2019; Plangger and Montecchi, 2020). However, consumers are increasingly demanding that the methods used by organizations that influence and affect their behavior are exposed (Heimstädt and Dobusch, 2020), that organizations or other entities build new systems utilizing the privacy-by-design ethos (Romanou, 2018; see also the Internet Freedom Foundation) and that, subsequently, users gain a greater level of control over their personal information (Boerman et al., 2021). The latter could include taking ownership of one’s own data, although this brings with it its own challenges without an appropriate regulatory support system (see Matz, 2021 for critique of user-owned data). We already see large tech companies starting to act on the demands from users and legislators for reduced tracking and monitoring, including Apple, who introduced the App Tracking Transparency pop-up which enables users of Apple iPhones to tell apps they are using not to track them. Now all apps with tracking behavior must include the App Tracking Transparency pop-up to gather users’ consent before enabling tracking (Chen, 2021). In a similar vein, Google has introduced a way to block third-party trackers in Chrome with an online advertising system called Topics, developed to prevent extreme tracking and sharing of user data with third parties that Google has built its business model on (Wakabayashi et al., 2022).

### ‘Social Life as Game’

There is a rich tradition of adopting “game theory” and “games” in the field of sociology. Swedberg (2001, p. 301) refers to this view of reality as “game-related sociology”. “Game theory” reflects a mathematical type of analysis that is applied to model how co-operation and/or conflict develop through decision-making over time, with an often featured example being the iterated prisoner’s dilemma (see, for example, Rapoport, 2016). “Games” on the other hand is often adopted as a metaphor for human activities that are played out according to a set of more or less explicit rules. Swedberg (2001) provides an insightful overview of how game theory and game-related analysis have been used in sociology. In this study, we are particularly interested in the application of “Games” as a metaphor to explore how individuals choose to interact with and respond to the actions of organizations in digital spheres with the view to manage their privacy and online visibility. Using “game” as a metaphor is helpful to direct focus onto the players who make their moves; the game setting and rules; and the strategies adopted to secure desired outcomes (Swedberg, 2001).

We are interested in the purposeful actions individuals take to open themselves up and heighten their visibility, while in other situations seeking to be closed off. We are interested to explore how individuals verbally account for these actions and what lies behind their decisions to heighten or lower their visibility. We know from other studies (e.g., Acquisti et al., 2020; Mwesiumo et al., 2021) that many users are interested in actively managing their levels of privacy online. While at this stage, it may only be a utopian dream to reach one’s desired level of privacy, there is an increased refusal amongst people to simply accept the rules that especially, big technology companies (e.g., Facebook, Amazon, and Google) create for users to play by and which are designed to make users part with their data and feed the construction of models of consumer preferences (Mulligan et al., 2020).

Goffman’s conceptualization of “game” is particularly useful to this exploration. In his book “Strategic Interaction” (1969), Goffman writes about the game-like nature that defines the interactions between actors who are aware of each other and where one actor’s move affects the move of the other. This highlights Goffman’s interest in and focus on the strategic interactions between knowing actors and the interdependence of their responses, choices and behaviors. Goffman elaborates on this game-like dynamic, which highlights the centrality of what he refers to as ‘moves,’ anticipation of potential outcomes and the, at times, opaque nature of the game being played (Goffman, 1969, pp. 149–150):

*“Persons often don’t know what game they are in or whom they are playing for until they have already played. Even when they know about their own position, they may be unclear as to whom, if anybody, they are playing against, and, if anyone, what his game is, let alone his framework of possible moves. Knowing their own possible moves, they may be quite unable to make any estimate of the likelihood of the various outcomes or the value to be placed on each of them. And bad moves often lead not to clear-cut penalties as such but rather to diffuse and straggling undesired consequences – consequences that result when persons do something that throws them out of gear with the social system.”*

Goffman uses the metaphor to illuminate the game-like calculations that individuals engage in when making decisions, especially when the loss of face is at stake, and to bring to the fore how information can be manipulated and controlled by actors in an attempt to craft an advantageous position in competitive interactions (Manning, 1991; Lemert and Branaman, 1997).

Goffman believes that, at most times, individuals will attempt to maintain what he refers to as the “ritual order” (Goffman, 1967), which means that they seek to preserve their social status and position. However, at times, individuals seek to change things, being willing to risk some of what they have for the prospect of winning ‘face’ and building on the self. This is what Goffman refers to as “fateful activity” (Goffman, 1961); activities that are potentially problematic and with consequences. These are also the conditions which foster enthusiasm and commitment to games; there must be something at stake, and there must be an opportunity to show off and display attributes that are considered valuable within the specific social context. For a game to be successful, there needs to be an element of uncertainty in terms of the outcome of the game (Goffman, 1961). The interest in the game is formed as the players go head-to-head and make their moves.

We focus specifically on Goffman’s metaphor of expression games, presented in his essay “Expression Games: An Analysis of Doubts at Play” (1969, pp. 1–83) since it is here that he most explicitly explores the “general human capacity… to acquire, reveal and conceal information” (p. 4).

### Expression Games

The expression games metaphor is used to illuminate how actors’ acquire, reveal, conceal and uncover information in their interactions with other actors, and seek to find out what the other actors might be revealing or concealing themselves (Goffman, 1969). These acts are often deliberate and purposefully executed to achieve a desired goal. Information sharing is controlled and manipulated depending on what is at stake from interacting in the game.

Situations arise when Actor A needs information that can only be provided by Actor B and not from any other alternative source. This can make Actor A dependent on Actor B’s willingness to reveal trustworthy and ingenuous expressions and information. Actor B may either be inclined to help Actor A’s assessment or may indeed make it difficult for Actor A to gather a truthful assessment (Goffman, 1969). Actors may choose to manipulate their expressions and the information that is communicated, making it difficult for other actors to know what to take at face value and what to be suspicious of. This highlights the game-like conditions and dynamics that are central to expression games. The different actors interact in a game to disclose and withhold information and expressions, trying to anticipate the other’s next move. At the core of expression games is the manipulation of information and the interactive ‘dance’ between the involved actors. Goffman (1969, p. 3) explains:

*In pursuit of their interests, parties of all kinds must deal with and through individuals, both individuals who appear to help and individuals who appear to hinder.*

In these game-like conditions, the game is played and progressed through the utilization of ‘basic moves’ (Goffman, 1969). These basic moves include the unwitting move, the naïve move, the control move, the uncovering move and the counter-uncovering move. For this study, we focus most specifically on the control move. The control move is used by actors to intentionally create expressions, which the actors believe will better their situation if gathered by other actors. Hence, this is a calculated act and central to the game-like nature of expression games. Goffman (1969, p. 13) explains that a control move “is made relative to a world that has already been generated by the game.” So, Actor A knows and understands that the immediate environment, including himself/herself, will give information to Actor B. Instead of being passive, Actor A seeks to influence how Actor B interprets this immediate environment and the information transmitted. Hence, Actor A engages in a form of role play by taking the perspective of Actor B when assessing their own activity. This enables Actor A to attempt to take control of the situation and anticipate how Actor B may approach the interaction and therefore how a response should be prepared.

Within the basic control move, actors have three types of moves they can play: concealment or cover, accentuated revealment, and misrepresentation. For this study, we focus specifically on the concealment or cover move, which Goffman states is also the most important move to explore (1969, p. 14). This move can take manifold forms: open secrecy and privacy, postponed decision-making and action, randomization, covert concealment, minimized sharing and feinting and feigning. It was important for us to identify the specific types of strategic actions and responses that actors can adopt to explore in detail what our research participants actually *do* to manage their online visibility. In our analysis, we draw primarily upon Goffman’s notions of covert concealment, minimized sharing and feinting for these are most pertinent to our overarching theme of exploring how actors knowingly manage their visibilities online. Covert concealment refers to the use of secretive signs and other forms of hidden communication. Minimized sharing is another approach subjects can take when they wish to be careful about who information is shared. They may seek to limit the number of people and observers who have access to the information and the amount of information accessible to them. Feinting refers to how subjects pretend and fake to confuse and create uncertainty in the mind of the observer. Feigning refers to “beliefs, attitudes, and preferences misrepresented strategically” (Goffman, 1969, p. 16).

In what follows, we seek to understand more about how our consumer participants knowledge of this context in which and through which more and more of their activities take place and develops. And, perhaps more crucially, how that increasing awareness impacts upon their knowing practice in and around digitized spaces.

## Materials and Methods

This study adopted a qualitative approach to exploring how users of digital platforms, devices and technologies interact with organizations in online spaces. The aim of the study was to explore in-depth the experiences, behaviors and actions of individuals who spend much of their time online. We seek to empirically identify and demonstrate how these interactions take on game-like characteristics as participants find ways of both revealing, and concealing information to protect their privacy and lower their digital visibility in encounters with organizations.

### Research Participants and Sampling

The primary focus of this study was to explore if those who are native users of digital technologies, devices and platforms take purposeful and calculated action to influence and control the level of accessibility organizations have to their information. While the focus was on the actions individuals take, we were also interested in capturing participants’ experiences of interacting with organizations in digital spheres and their understanding of how organizations acquire and make use of their information and data.

We conducted in-depth interviews in combination with photo-elicitation with 20 purposefully selected individuals (thirteen females and seven males) who are experienced users of digital technologies and native to digital consumption, communication, and content creation. Our participants are in the age group 21 to 26 years old (born between 1994 and 1999) and have completed an undergraduate university degree in the United Kingdom (UK). All participants are now in employment and are based in different parts of the world, including the United Kingdom, the United States, Europe, and South America.

While our participants were purposefully selected to ensure that they are active users of digital platforms and have been exposed to digital technologies from a young age (e.g., ecommerce platforms, online entertainment and music, social media and messaging, online learning and other digital conveniences included in the on-demand economy), we would not consider our sample an ‘extreme sample’ whose experiences are completely unique to them. Having said that, we also recognize that other individuals, with other characteristics may articulate their experience otherwise in relation to the themes of our interest here.

### Data Collection

**FIGURE 1:**
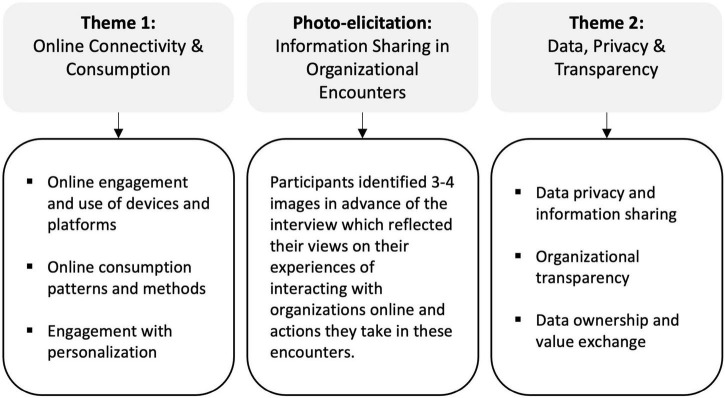
Design of interview guide and photo-elicitation.

The in-depth interviews were conducted with participants via a video conferencing platform and lasted on average 80 min. The interviews were guided by a semi-structured checklist of areas, which was informed by our research focus. Structuring the interviews assisted in the cross-participant comparison (Irvine et al., 2013). The interview was divided into three parts (Figure 1). The first part focused on exploring participants’ digital connectivity and use of digital technologies to interact and engage with organizations. We were interested in their online consumption behaviors, their views on and experiences of organizations’ attempts to personalize their communications, offers, content and products/services to their individual needs as well as how they navigate and make decisions on what information to share and when to share this with organizations. In the second part, participants were invited to share the 3–4 images they had collected as part of the photo-elicitation pre-interview exercise. Each participant had chosen their own images as a way of capturing their experiences of and perspectives on interacting with organizations in digital spaces. Participants shared the images in real time via screen-sharing functionality and used them as visual cues and prompts for their accounts. Incorporating photo-elicitation as a supplementary method alongside the semi-structured interview proved highly effective in enabling participants to start the reflective process prior to the interview. We were conscious not to provide participants instructions that would restrict their choice of imagery but kept it quite open to create space for participants to identify their own narrative, reflective of their specific and individual views and experiences. During the interviews, we noticed how participants became excited when asked to share their chosen images and were keen to explain why they had chosen them and what they represented. This transition from participants being interviewed to participants taking the lead in the conversation meant that participants were not only receiving questions to respond to, but were also given the opportunity to share the output of their preparation. In the interview, it was evident that the images provided participants with useful support to explain thoughts and experiences that, at times, were complex and fuzzy, but gained clarity with the visual imagery (Warren, 2005; Davison et al., 2015).

Hence, We saw how the use of photo-elicitation can give voice, empowerment, and agency to participants as they choose which images to select and how to present and contextualize them (Heisley and Levy, 1991; Harper, 2010).

Table 1 provides an overview of some of the images that were shared with us by participants. While some images were used as metaphors (e.g., Images C–G), others directly referred to events that had recently taken place (e.g., the Cambridge Analytica scandal; Image B) or were used to make cultural references to popular movies and TV programs (e.g., Black Mirror and The Circle) that focus on issues related to data privacy and digital visibility.

**TABLE 1:** Examples of images from photo-elicitation.

Image A 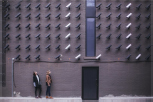	Image B 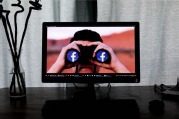
Image C 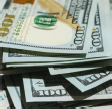	Image D 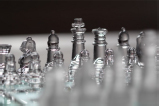
Image E 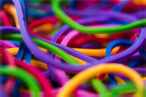	Image F 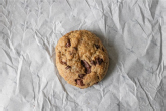
Image G 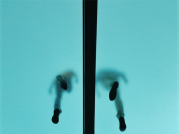	

*Source: All images are sourced from unsplash.com.*

It was essential that the interviews captured participants’ authentic experiences and that the questions did not lead participants on. As such, the questions purposely did not ask participants in direct terms about their ‘game-like’ behaviors and actions. We were conscious that if the game-like behaviors and actions exist, they first and foremost needed to emerge from participants’ descriptions and accounts. Hence, the insight that emerged was participant-led, facilitated by the questions in the interview guide and the photo-elicitation element. Participant quotes have been anonymized via the use of pseudonyms.

### Analysis

**FIGURE 2:**
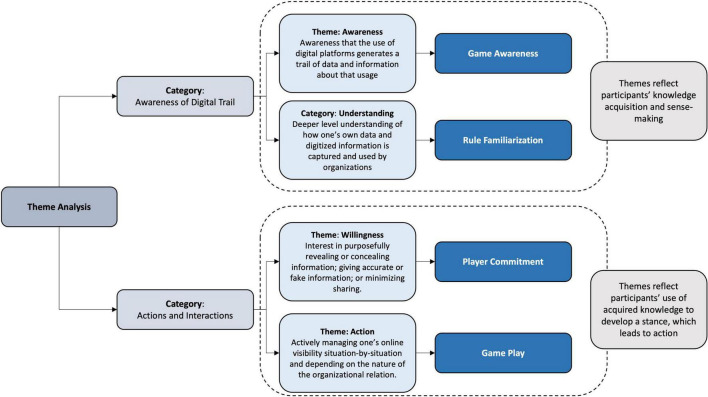
Theme development.

All interviews were recorded and transcribed verbatim. We started the analysis by coding the interview transcripts according to two broad categories. The first category contained our observations related to how participants express their awareness of how they generate data and information when they use and engage on digital platforms (we refer to this as ‘Awareness of Digital Trail’). The second category was focused on participants’ actions, i.e., what participants do, when they use digital platforms and, specifically, when they interact with organizations and platforms owned by organizations (e.g., social media platforms) (we refer to this as ‘Actions and Interactions’). In our deeper analysis of findings within these two categories, we evidence more nuanced and detailed observations pertaining to how our participants navigate their existence on digital platforms, what knowledge they have of how their information is generated, how they develop their stance toward organizations’ desire to acquire their information, and the types of moves they make to reveal or conceal their information. In Figure 2, we summarize the process that took place toward the presentation of the final themes that led to the creation of the P2G framework.

Within the category ‘Awareness of Digital Trail,’ it was clear that participants had awareness of how user activities on digital platforms, in general, generate a digital trail of data and information. However, we also observed that participants applied this awareness to their own lives as digitally connected individuals and that they understood that they also make many decisions on a day-to-day basis about what information to share and with which organizations. It was evident that participants have a rich understanding of the dynamics that exist in digital spheres and how organizations go to great lengths to encourage users to share their information with them, often with the promise of an enhanced and personalized service, product or experience in return. As an outcome of this phase of our analysis, we identified the themes ‘Awareness’ and ‘Understanding.’

Within the category, ‘Actions and Interactions,’ the data pointed to what participants do to manage their online visibility and interactions with organizations. However, we also detected a state that precedes this where participants are weighing up what they stand to gain and what they may lose when revealing their information based on organizations’ explicit or implicit requests. Participants make such considerations according to the specific situation and based on other contextual factors, including trust in the organization and the amount of effort required to circumvent organizations’ digital information accumulation structures. Based on this more detailed analysis, we identified the themes ‘Willingness’ and ‘Action.’

In the final phase of the analysis, we re-engaged with the data and themes through the lens of Goffman’s expression games metaphor and the notion of game-like behaviors and responses. Adopting this theoretical perspective enabled an abductive analysis (Lewis-Beck et al., 2004), which is an approach used to construct descriptions and explanations based on everyday activities in an attempt to understand individuals’ actions, motives, and rules. This analysis made visible the game-like nature of interactions that take place between our participants and organizations. In line with the game metaphor, the final set of themes reflect the game dynamics and the states that participants engage with from *Game Awareness* to *Rule Familiarization*, to *Player Commitment* and to *Game Play*. We present these states as part of the ‘Propensity to Game’ (P2G) framework in Section “Results.”

## Results

It became evident from the research that managing one’s online visibility is of importance due to concerns over mass surveillance and data-driven manipulation outside of participants control and influence. However, to be a vigilant digital game player requires much effort to continuously learn about the methods and techniques used by organizations to create structures and mechanisms for information capture. The lack of transparency has led to ‘digital minefields’ with traps and hidden implications that participants at times struggle to navigate through due to the opaqueness of future possibly consequences. Thus, participants often lack trust in organizations and they assume a position of wary caution. From their accounts, it is evident that they engage in ongoing learning processes to acquire the necessary knowledge to understand and respond to the changing methods organizations use as part of their attempts to heightening users’ visibility.

**FIGURE 3:**
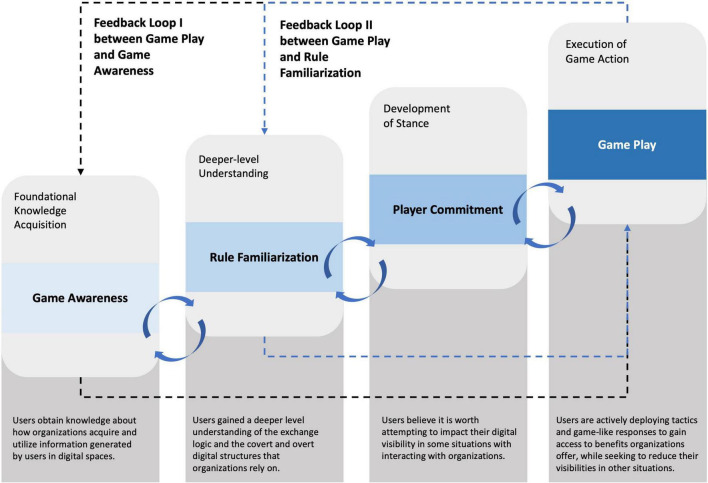
‘Propensity to Game’ (P2G) framework.

Based on the insight we derived from our investigation we present the P2G framework (Figure 3) which depicts four states (*Game Awareness*, *Rule Familiarization*, *Player Commitment*, and *Game Play*) that users engage in as part of their game-like interactions with organizations. Typically, a user first needs to acquire an awareness of the game (i.e., that users’ information generated through their actions and behaviors in digital spheres is of immense value to organizations and that organizations are keen to make users as visible as possible for information extraction) before the user may obtain a deeper level understanding of the specific rules that define the game (i.e., the user now observes how their information is captured by organizations and how they are affected when they share information about themselves). Once the user has acquired *Game Awareness* and *Rule Familiarization*, the next state, *Player Commitment*, reflects the user’s willingness and interest in playing the game and becoming an active participant (i.e., the user may be more or less willing to adapt their online behaviors and actions to proactively manage their online visibility). If the user is interested and willing to play the game with organizations, they progress on to the final state, which is *Game Play*. At the *Game Play* state, the user is acting on the awareness, understanding and willingness accumulated from the previous three states and converts this into specific and calculated behaviors that are used to knowingly and intentionally create expressions that the user believes will better their situation if gathered by organizations. Importantly, although the states in the framework are progressive, together in practice they appear to form something akin to a faulty ratchet. Whilst engagement with ‘higher’ states seemed to require prior passage through ‘lower’ states, myriad contextual elements often could and did conspire to drop participants back down to interaction marked by lower state characteristics. The P2G framework also incorporates two feedback loops (I and II) which illustrate the dynamic logic of the framework and the continuous work required by users to understand new methods and techniques organizations use. Some users therefore re-engage with the Game Awareness and Rule Familiarization states to ‘top-up’ their understanding and knowledge of the evolving digital realities.

### Game Awareness

The first state in the P2G framework is *Game Awareness*. *Game Awareness* reflects a participant’s basic realization that their activities on websites, through mobile apps and other digital platforms generate a digital trail of information about their behaviors, actions and choices they make. Hence, at the *Game Awareness* state, participants have realized that they always engage in exchanges and transactions with the organizations they interact with; their information being part of what is traded. Importantly, participants who have acquired *Game Awareness* have also realized that much of the information they share with organizations is of commercial value and that they are not always in a fully agentic position to decide what to share and what not to share; let alone deciding when to be seen and when to achieve a lower level of visibility. Users with *Game Awareness* acknowledge that organizations are not fully transparent about their business practices, in particular how they acquire, assess, analyze and use users’ data and information. Thus, *Game Awareness* refers to having awareness of the basic tenets of the digital economy where organizations and consumers engage in an interplay of information creation, sharing, acquisition and concealment, central to many organizations’ business models and essential for consumers to have access to many digital services, platforms, and experiences. Our participants were aware that their data is critical as a competitive asset for many organizations due to its value:

*Everything you sign up for now, everyone tries to get data out of you. And after a while, you sort of realize there’s value in that data. (Marcus)*

There was a sense that the interactions between participants and organizations online are mediated by a ‘layer’ that prevents fully open and transparent communication between an organization and users from taking place. Jenna explained how companies, in this form of mediated communication, choose what to reveal, what to share and what information to make available to the user, reflecting her awareness of the basic game-like dynamics where information may be concealed or revealed depending on the objective of the organization:

*There’s always a layer between you and the company. It is never like there is ‘nothing’ between you and the company. It is never open communication. There’s always information that the company chooses to be there and chooses to share.*

The accounts of several of our participants revealed a tension in their relationships and interactions with organizations. Participants displayed mistrust toward organizations and the practices they adopt due to a lack of confidence in their intentions and a sense that they do not always have the users’ best interests at heart. Paula shared how she believes some organizations may be concealing information and business practices in privacy statements, which they know users are unlikely to read in full or even fully understand. This makes it challenging to make a truthful assessment of what one is agreeing to:

*I think probably the way that people are so quick to accept the privacy statements might benefit the companies. There might be things in those agreements that companies don’t necessarily want consumers to read or know.*

Participants described how they feel the need to be careful and cautious when interacting with organizations as they have the sense that there is always something else going on in the background, which they do not have full sight of. There was a sense that they are interacting in spaces that lack transparency, where they do not have full autonomy to make their own decisions and where organizations have created digital infrastructure and systems which they become a part of without ever fully knowing what they have agreed to, what information they are sharing and with limited power and authority with which to challenge them. James shared his concerns about how the data generated from his online activity never becomes fully visible, but is concealed within organizational structures:

*You can never see, you don’t get to see a database on yourself. You don’t know what data the companies have collected about you. When you have no idea what information has been collected about you and how this is being prioritized by companies to create your customer profile, then this is what makes it unsettling.*

At the *Game Awareness* state, participants have also become aware that their previous search and purchase data inform the recommendations that organizations present them with in the future. They have an awareness that decisions a user has made in the past are never forgotten in digital spheres and that this information is of great value to organizations, who will use this for future predictions and construction of preferences. The *Game Awareness* state revealed that participants have become aware that Big Tech, such as Facebook, Twitter, Instagram, and other social media platforms, acquire users’ data as part of their business models to create economic value for themselves. There was a sense that these organizations are profiting from individuals’ use of time building these platforms, which initially were positioned as social spaces for connecting with friends. Participants recognized that these platforms have created extremely powerful and dominant companies due to the rich predictive resources that they have acquired.

To sum up, the state of *Game Awareness* reflects participants’ basic realization that digital spheres are not neutral and without tension. These are spaces where organizations and users engage; sometimes driven by the same, sometimes differing interests, which form their interactions and users’ moves toward managing their visibility.

### Rule Familiarization

*Rule Familiarization* reflects participants’ deeper level of engagement with the game-like conditions in digital spheres. Participants have realized that organizations are not always truthful or transparent about their intentions and practices and that dark patterns are employed in the design of digital interfaces that lead them to share information which, if given the choice, they would rather not reveal. *Rule Familiarization* is characterized by how participants ‘activate’ their *Game Awareness* by relating this broader insight to their own online lives and thereby adopt a more introspective standpoint. Now the question is “How am *I* affected by these organizational practices?” Participants realize how they themselves are interwoven in tactical game playing with organizations in an attempt to manage their online visibility and information sharing.

Central to the game-like conditions was how participants frame the dynamic interplay between themselves and organizations as *‘them and us/me.*’ There was often a strong combative undertone in participants’ explanations of how they navigate digital interactions with organizations based on mistrust and suspicion and a familiarization of the rules that guide these interactions. Emma shared her understanding of some of the tactics organizations use to acquire users’ information. More importantly, she revealed how she knows that organizations influence her decisions and that these are not always in her best interest. She at least would like organizations to pretend that she is empowered to act on her own wishes:

*I’m definitely not going to click the ad, which appeared on my phone 5 secs after I said something. Again, you’re targeting me, you might have already scanned my phone and the apps that I have on the phone and figured out that I would be the perfect consumer and I would actually like your product. I don’t know about other people, but I personally at least want to have an illusion of making my own choice.*

This sentiment was also expressed by Willow, who shared how access to information made available by organizations often has ‘strings attached’ and that to get this information, users must exchange their data in return. She believes these rules of engagement may be changing as she has experienced more organizations being open about their data collection practices:

*Wi-Fi networks will be like, “Can we use your data?” Um, I think that that’s something where a lot of people just default, like, accept without truly understanding “Okay, what does that mean? Who is using my data?” So, I think that there’s a good movement toward becoming more transparent with things like this. There are always strings attached if you want to get access to something.*

It became evident that participants give much thought to what kind of information they wish to share with organizations and that, in their view, there are different categories of information in relation to this that they would wish to have treated differentially. Some information is deemed non-sensitive, and participants are less concerned about sharing this, while other types of information are deemed to be of a more personal nature. This illuminates how participants engage in on-going reflection and categorization of information, deciding on a case-by-case basis which information they are willing to share and with which organization. It was not uncommon for participants to question why organizations need access to certain types of information. This highlights how participants challenge the rules that have become central to how they interact with organizations. Jenna’s experience exemplifies this on-going negotiation and decision-making related to what information to reveal and what to conceal, highlighting the dynamism of managing one’s online visibility:

*So, I think that there are some general things that it would be normal for some companies to have access to, so maybe your email address, maybe your first name, your last name. I don’t necessarily know why that would be relevant in some cases, especially if it’s just for advertising. And if you’re a female or male, in some cases, it might be relevant. But then, again, like in other cases, why do the companies even care? And then location, I think location is something that bothers me if they ask for my location, or my postcode, I feel that’s a bit intrusive.*

It emerged that some rules that define the game-like conditions are easily verbalized by participants, while others are fuzzier and more difficult for them to explicate. For example, if participants shop online, they know that companies are going to capture that shopping data and use it to understand their buying habits. This has become a normalized exchange where shopping data is shared with the organization in return for convenience, online product access and, at times, personalized recommendations. In these situations, they understand that their visibility as a ‘shopper’ is heightened and that many organizations rely on this information to understand consumer trends and (construct) consumer preferences. However, the rules that define the game were not always observable by participants. Participants expressed how they believe organizations are using their information in ways which are not transparent or openly communicated to them and consequently they cannot anticipate the consequences of sharing the information with the organizations. As such, they cannot anticipate the organization’s next move and therefore struggle to proactively manage the extent of visibility. Participants used different expressions to describe these situations, such as organizations having ‘a different interest’ (Sophia) and that they can use the information as a ‘weapon against me’ (Mia). In the quote below, we hear from Emma:

*With those long scrolls, and when I need to turn everything off, you know that nobody is going to be bothered to do this. So you’re just using me as a guinea pig sitting in front of the screen, just waiting for me to give up and allow you to collect all my data. Maybe they don’t think like that. But this is how I feel. Oh, and again, in every single relationship, you have to have respect and every single business relationship, you give something you have something back and you don’t like when something is happening behind your back and you’re not aware of this. (Emma)*

It was evident that participants feel uncomfortable about not being able to obtain a full understanding of how their information is used by organizations and how this information may affect them in future encounters. Participants described how they always feel ‘observed’ and that some organizations force them into a constant state of online visibility. In these situations, participants have little choice about whether they wish to share this information or not and the information they generate is inherent to their use of the digital platform. Hence, the information trail they generate cannot be separated from their use of the platform. Here, James describes this intensified level of surveillance he experiences, for example when he is spending time on Facebook’s social media platform:

*I was thinking about Facebook, for instance, and it just feels like they are watching you through your screen. And they’re collecting data from your screen. And it’s a bit scary to think that if you put your mouse somewhere on the screen, they can realize how many seconds you’re sitting in one place or another. And they know if you’re sad of if you’re happy and how that affects your consumption.*

Across participants, there was a desire to challenge the existing rules that define their online activities. In their views, the new rules of engagement between organizations and users should be based on a shift in ownership of data and information from organizations to users. Participants expressed that this shift would enable them to reclaim power over their information and have agency to decide who this information should be shared with. This would significantly alter the rules of the game and enable users to actively influence the assessments that organizations are able to make about them, their behaviors, actions, and future aspirations. Casper shared his ideal scenario where users own their data, and in that ownership get to decide who the data is shared with, if any:

*It feels like your data should be yours and that it should be your right as a human to own your own data. I would personally be excited if we could live in a future where we would be able to have our own data secure and own our own data, which sounds very ironic thinking about it that you don’t own it right now, just because somebody started it, and you were unconscious about it. And now it’s so hard to stop it, because it’s like, a roller coaster that keeps on spinning. And it’s like, you can’t really stop it and say, ‘Okay, now that’s it. I’m not going to share anything anymore.’*

Importantly, the explicit desire for this paradigm shift also points to the nature of the current game-like conditions: participants believe that, despite their awareness and understanding of the game-like dynamics and their ability to take action to lower their visibility, organizations still possess the upper hand.

To sum up, the state of *Rule Familiarization* reflects participants’ deeper level of engagement with the game-like conditions in digital spheres. There is a realization that they themselves are part of the game and affected by organizations’ access to the information that they generate.

### Player Commitment

*Player Commitment* reflects a participant’s commitment and willingness to adapt their online behaviors and actions. This reflects a proactive position where participants are interested in how they manage the sharing of information with organizations and consequently their level of online visibility. *Player Commitment* reveals that some individuals do not only possess *Game Awareness* and *Rule Familiarization* but are also actively exploring opportunities to engage in more tactical information sharing, feeling that there is something ‘at stake.’ Several participants shared how their commitment to act on their awareness and understanding of the methods organizations use to capture their information have given them an active role in deciding what to share.

*You very consciously uncheck boxes now. Whereas before, you’d be like, “Yeah, tick it and leave it.” But now you actually read what they’re asking. I think it’s more a case of taking an active role in what you put out there and what information you’re sharing. It’s an exchange of information rather than just them providing a product to you. (Marcus)*

The intensity of *Player Commitment* varies from one individual to another. The intensity level depends on several factors, including what the individual believes they can gain from sharing their information with the organization; the level of trust the individual has in the organization; and the amount of effort required to make oneself less visible.

#### Information Sharing Versus Perceived Gain

When participants considered whether to share information with an organization (when this is a conscious and active choice), they often decided on the basis of what the organization will share with them in return and the value of this to them. This could include information about an upcoming sale, information about new product releases, discount codes and personalized product and service recommendations. When participants believe it is worthwhile to share their information, they seek to heighten their level of visibility and show more of themselves. This transaction-based negotiation was accounted for by most participants, including Ben:

*I think I am self-conscious about what I give out. I don’t want there to be a huge amount of information that I give to companies. And I don’t want companies to know and to feel like they are tracking me. I feel like if it’s a two-way street, I’m willing to give a certain amount of information for a service that they can provide me.*

This, however, is often not a sustained and continual visibility. Often, participants choose to lower their level of visibility if they deem that the value organizations create for them is no longer of interest or worth the information given in exchange. This is seen, for example, when participants share their information to subscribe to an organization’s newsletter with deals and offers or to take part in a competition and then purposely unsubscribe as soon as they have reaped the benefits.

#### Trust in the Organization

Trust was an important factor in influencing participants’ willingness to lower or heighten their visibility. When participants trust an organization, they believe it is less likely that the organization is intending to misuse their information, manipulate them or in other ways use the information to act unethically. Mia explained how she is open to sharing her information with an organization which she trusts. She also acknowledges that, in her experience, not all organizations can be trusted:

*I don’t mind organizations knowing my information. It’s just a matter of trusting that they can do the right thing with it, which is not always the case.*

The identification of organizational trust is an on-going process and often one that is considered on a case-by-case basis as participants re-engage with organizations and encounter new ones. It was evident that trust in an organization is built over time and from having continuous interactions without participants feeling that the organization misused their information. Several participants appreciated organizations that sought to provide users choice about sharing more or less of their information (e.g., by allowing individuals to easily ‘Reject all cookies’):

*If it’s a company that I know something about and they are quite transparent about what they are doing and how they collect data, then I trust them with my personal details, and also because I know that I’ll be using their services. But if it’s just like a website, that I randomly found and I’m not really sure if it’s going to be relevant to me, then I don’t really trust that company.*

The influence of trust illuminates how participants are selective when they decide whether to share information. They may choose to share certain information with one company, but not share the same information with another. In these situations, they exercise minimized sharing by being careful with who information is shared and the amount of information.

#### Required Effort to Manage Visibility

It became evident from participants’ accounts that managing their online visibility does not come without effort. It requires a dedication of time to develop the *Game Awareness* and *Rule Familiarization* and consequently develop at least some extent of *Player Commitment*. With the presence of *Player Commitment*, participants now need to understand what one can in fact do to manage one’s online visibility in a variety of different situations and encounters. While participants expressed a desire to more actively control what information they share and reveal, this can be challenging in practice due to the complexity of interconnected digital systems and use of tracking and surveillance methods, which are often not apparent or transparent to them. This, therefore, demands even more effort from participants to identify the ‘information dials’ and develop an ability to foresee consequences of their calculated actions. In a way that represents the experiences of most participants, Ben shared his perspective on the complexity and the difficulty of understanding what the effects of one’s actions in digital spheres are:

*It is such a complex, complex world out there and everything’s so intrinsically tied together, and it’s all so knotted up. It’s actually very hard to break down to what level you are giving information. When you’re giving the information, what are the full consequences of it? I don’t think anyone quite understands that or knows that, myself included. I don’t know, for example, if someone’s else is recording the call or listening to me when I speak and so on. And actually, you know, I don’t understand, necessarily, what information does get used when I give it. You know, are they tracking those trends and therefore able to see what I’m interested in? It’s really difficult to understand what the consequences are and to fully understand what the complexities are.*

Despite the desire to become more active players in the management of their online visibility, several participants also felt that it may be too late to go up against organizations in this game of differential information accessing and sharing. There was a sense that the effort required to truly manage what organizations see and what is concealed is beyond what an individual can do and that even with a dedicated concealment effort, organizations will somehow gain access to their data and information. Like several of the other participants, Emma is very game aware and understands the mechanisms she can use to conceal information. However, coupled to this understanding was also a feeling of powerlessness in the face of the extensive digital infrastructures already in place:

*From everything that has happened and everything that I’ve read, and everything that I know, I feel quite powerless and a bit pathetic and I want to protect my data. But I feel that if I wanted to keep it all to myself, I should have started putting a proper effort into it and constantly be maintaining the security settings. So, forget about the fact that most of my efforts are most likely futile.*

Although participants felt that organizations make it difficult for them to opt in and out of information sharing, they also expressed a keenness to learn and adapt their behaviors nonetheless. Participants reflected on behaviors they adopted in the past when they were less knowledgeable about the rules, game dynamics and the value of their information and compared these past behaviors to their more informed actions of today. Here, Emily shares her discontent with allowing third party companies access to her information and how she has acquired ways of minimizing sharing with companies of this nature:

*It’s very, very key not to ever share your information with third parties. That is very key. And obviously you have to tick, “Do you agree to that or not?” So, maybe in the past, I haven’t thought about it, and I have clicked the wrong thing, and it’s my fault, but I cannot imagine why would anyone want to have their data shared with a third-party company willingly. There is no benefit to you from it apart from 10 years down the line, you’re gonna get some sort of scam call.*

Whilst all participants expressed an interest and desire to become active players in the games they play with organizations, there is also no doubt that this requires significant time and effort to keep up to date with changing security settings, understand the role of third-party companies and their accessibility, and be able to anticipate how an organization may attempt to use their information for undesirable purposes.

In summary, *Player Commitment* is an indicator of an individual’s interest in the game, which also includes challenging the rules and seeking to redefine how the game is played. Participants who expressed a pronounced commitment to playing the game with organizations also felt that there is a need for organizations to become more transparent, share more information and be more open about what they want from individuals and how they intend to use individuals’ information. This points to, not only a change of the rules, but a rearrangement of the dynamics to reduce the level of uncertainty, concealment, and manipulation, which participants often feel organizations use as game tactics.

### Game Play

At this final state, participants are acting on the *Game Awareness*, *Rule Familiarization*, and *Player Commitment* they have accumulated. They are now focused on putting this into action through specific and calculated behaviors used to intentionally create expressions that they believe will better their situation if gathered by organizations. In other words, *Game Play* reflects the actions and behaviors individuals construct with the purpose of influencing how organizations access and interpret the information that individuals transmit.

In this research it was evident that participants make attempts to take control of their interactions (i.e., deploy *control moves*) with organizations and shape these interactions for their own benefit. Participants’ behaviors and decisions are not completely random and spontaneous acts, but often calculated and deliberate decisions made in each interaction and based on their assessment of the organization and what ‘moves’ the organization may respond with. This interest in anticipating and understanding how organizations behave and respond to users’ actions is central to *Game Play*.

Participants expect organizations to initiate interactions with them, and they have also understood that these interactions typically require them to reveal information about themselves, their interests, preferences, and aspirations. This is a highly dynamic interplay where situations evolve and unfold depending on the moves made by the users and the organizations. To better anticipate how an organization may react and respond, participants engage in a form of role play by ‘thinking like the organization’ and viewing the interaction from the organization’s perspective. When this perspective is adopted, participants are able to better identify what an organization is looking to acquire from the interaction, why the organization may conceal certain information and what may likely be its next move. Participants shared how they ‘play out’ the scenario of what is likely to take place before it happens and use anticipated scenario to make decisions about what to share based on how they would like the scenario to unfold. This is not to say that there are no blind spots in their assessments.

The type of control move reported as being mainly adopted by participants in this research is the ‘concealment or cover move.’ Within this move, there are multiple tactics that can be adopted to manage one’s online visibility. There was evidence of participants engaging in covert concealment, minimized sharing and feinting as presented in the subsequent sections.

#### Covert Concealment

This tactic is used in interactions where it is deemed necessary to use hidden communication or secretive signs to conceal the information shared from one or more parties. Goffman (1969, p. 14) refers to the use of “a mask or camouflage of some kind” to further blur the information that is being transmitted. It was evident that participants make use of the covert concealment tactic when they wish to lower their visibility and undertake tasks and activities out of sight of certain organizations. The tactic was put into practice when participants purposefully disguised their online search behavior and interests by accessing Virtual Private Networks (VPN) which allow users to extend a private network across a public network and encrypt information that is sent and received. Using a VPN to conceal the information that is sent and shared acts as camouflage for participants to prevent organizations from capturing the data they otherwise generate when they access websites, type words into search engines and make purchases online. In a similar vein, some participants sought to hide their communications by using the Tor (also known as The Onion Router) Browser when searching for information. The Tor Browser prevents third party companies from tracking users, limits advertising, and cookies are automatically cleared after each session of browsing along with the user’s browsing history. The Tor Browser was primarily used as an alternative to interacting with the Google Chrome Browser, which participants know collects and analyses their search patterns and input, leading to individualized search results. Not all participants want Google to ‘tailor’ the search results based on their previous searches and websites visits.

#### Minimized Sharing

Minimizing sharing is another tactic used by participants to manage their visibility and influence how organizations interpret their behaviors and actions. This specifically entails being careful about who information is shared with and limiting the extent of access and the amount of information shared. Participants made use of the minimized sharing tactic when they were concerned about the motives and practices of an organization, or the digital service offered. It was evident that participants do not simply share what is requested of them, unless they deem it relevant or are confident that the organization will be able to create an enhanced service or experience based on the shared information. A common situation that participants described was when downloading a new mobile app. While this is a relatively mundane act, participants explained how they are often prompted with requests from the app owners during the downloading process to gain access to the phone’s camera, photos, audio, and GPS location. In these situations, participants are having to decide what level of access to grant and consequently how much and what kind of information to share with the owners of the app. It was evident that participants are critical toward requests considered to be unrelated to the functioning of the particular app and will seek to only grant access to the necessary information needed for them to use and benefit from the app. John shared his experience:

*There are some apps that, okay, it makes sense for them to have access to the pictures or to camera stuff, especially if it’s something social media related. But with location, I’m always “Why would you need that?” I get that sometimes you get personalized recommendations based on your location. But then I’m a bit worried why each app would need my location. So, what I do is if I judge that the app doesn’t help me if it has data of my location, I just don’t allow it. But then for other apps, like even Google Maps, I put it on ‘allow only while using the app.’ So, I know that if I’m exiting the app, I have the peace of mind that it’s not going to track my location. But I’m also like, “do they really switch it off?” I don’t know. I have my trust issues relating to that, but I do choose to allow location tracking only while using the app and other apps I don’t allow it at all.*

The use of the minimized sharing tactic was also used in situations when participants had low expectations of an organization’s ability to use their information to create an enhanced experience. As an example of this, Zoe explained how she is happy to share her user data with a company such as Spotify as she believes her interactions with the music streaming service improves if they are able to understand her music taste and listener preferences. Whereas she tends to sign in as ‘guest’ on online shopping platforms as she does not see the value in the ‘personalized’ recommendations companies such as H&M offer her. Consequently, she chooses to minimize her digital information trail on these platforms.

#### Feinting

The tactic of feinting refers to the act of pretending and faking with the objective to confuse and create uncertainty in the minds of others. It is a tactic used to mislead or misguide through the sharing of information which is untruthful or inaccurate. Participants adopted this tactic in situations when they needed quick and often one-time access to information or services offered by an organization which they did not wish to continue an affiliation with. In these situations, participants purposely shared fake information of reduced value to the organization and were explicitly calculating in how they chose to represent themselves. This tactic was, for example, enacted in the use of multiple email accounts for different purposes. Most of the email accounts had been set up by participants with the main purpose of using these to extract information and services from organizations (e.g., when signing up for a newsletter or deals) without giving organizations their actual email address in return. Emma explained how she manages this in practice:

*I have six email addresses that I have access to. Yeah, and they’re all six in Gmail, one on Hotmail. So, for the very shitty websites, I give that mail. If it’s something related to, if I’m placing an order online on a website that is going to be a one time thing, I use this email. If I give my email for job purposes, it’s my main email and if it’s some kind of newsletter, it’s another email. (Emma)*

Participants also made use of the feinting tactic when accessing public Wi-Fi by providing incorrect names and email addresses as they had realized that access to Wi-Fi would still be granted. There were also accounts of how participants input inaccurate information when setting up online accounts (e.g., name and birthday and other qualitative information related to interests and preferences), despite or perhaps because of how this may mislead the organization and their profiling of users.

*If it’s free Wi-Fi in the airport, or in a coffee shop, I’m definitely not using my email, just because I don’t know, I feel like I have the option of just inventing something. And that works. And even for some websites, if they ask you to sign up, because it’s for free. If it’s something that I’m going to use on a regular basis, like for instance, there’s Canva, which is an online editing app, then for that I signed up with my own email, because I know that I’m using it professionally, but for other websites that I’m just checking sometimes, I’m not using my real email, so I’ve found a way around it without actually using your own persona and identity. (Sophia)*

To sum up, *Game Play* is users’ enactment of calculated behaviors that are used to intentionally create expressions that they believe will better their situation if gathered by organizations. We identified how participants use the control moves covert concealment, minimized sharing and feinting to influence their interactions with organizations and to lower or heighten their visibility depending on the expected outcome and organizations’ next moves.

## Discussion

Our work studies how consumers who have been well socialized as digital natives and who are extensive users of digital platforms, engage in dynamic and game-like interactions with organizations in an attempt to manage their level of online visibility and information sharing in relation, *inter alia*, to the ‘convenience’ and ‘benefits’ that are afforded to them. With this research, we seek to contribute to the growing literature on privacy and visibility management, including work done to understand consumers’ rational responses and the trade-offs they make between privacy protection and access to benefits (i.e., the privacy calculus perspective) (e.g., see Dinev and Hart, 2006), consumers’ attitudes toward surveillance activities and how this enables a categorization of consumer archetypes (e.g., Plangger and Montecchi, 2020), and the impact of perceived control on consumers’ willingness to disclose information (i.e., the control paradox) (e.g., Brandimarte et al., 2013). In particular, our research illuminates the game-like considerations and tactical moves that form part of consumers’ game play. Our findings demonstrate that these consumers are far from passive recipients of organizations’ communicative interactions. As has been evidenced in other studies (e.g., Acquisti et al., 2015; Bornschein et al., 2020) they are also not unknowing about how digital infrastructures are designed to enable organizations to reveal and acquire their data and information, often based on commercial interests (Flyverbom, 2016). As such, these consumers are aware that their actions in digital spheres leave behind digital trails and that many organizations are dependent on the information they generate to better the organization’s situation. They have become knowledgeable about how and when their information is acquired and used by organizations in the attempt to make reliable assessments of the nature of individual consumers as well as to group segments of consumers, which, in turn, influences organizations’ next move (e.g., content creation, product recommendations and advertising targeted to specific consumers based on past behaviors and predicted/constructed future preferences).

It is in this empirical context that the application of Goffman’s expression games metaphor and the three forms of concealment or cover moves, covert concealment, minimized sharing and feinting, are particularly helpful to illuminate how this awareness and understanding impact upon consumers’ actions. The gaming metaphor enables us to conceptually make sense of, organize and present the processual dynamics that characterize these consumers’ evolving engagement with organizations in the P2G framework. In our development of the P2G framework, we found the work of Acquisti et al.’s (2020) and their identification of consumer-organization interactions (e.g., information symmetries, bounded rationality, present bias, intangibility, and constructed preferences), which point to the action-response dynamics particularly insightful. Adopting a gaming perspective, our study contributes with further insight into how consumers act and behave in response to the anticipated interests and games played by organizations to extract their information. Goffman’s expression game metaphor and the associated moves to reveal, conceal or acquire information are effective in conceptualizing the game-like behaviors of our consumer participant group. Through this lens, our research presents a multitude of different situations where there is mutual awareness between the players (i.e., the consumers and the organizations) and where the move made by one player affects the other. As such, the P2G framework is a potentially productive mode of conceptualizing these consumers’ game-like interactions with organizations in a manner that contributes to our understanding of how this digitally native consumer group interprets, makes sense of, and responds to the increasingly contested value nexus that commercial capture, management, and exploitation of digital data entails.

The P2G framework adds new action-based insight into how, and in which ways, users navigate digital spaces by adjusting their visibility, making calculated acts, and anticipating organizations’ next moves. In this way, the P2G framework helps to illustrate how these consumers move from having basic and foundational awareness of how information is acquired and revealed to gaining a deeper level understanding, experiencing the impact of organizations’ practices on their own ability to manage their online visibility. Based on this acquired awareness and understanding, it was evident from the research that these consumers have reached a point where they feel the need to become active players in the gaming activities if they are to gain some level of influence over how their interactions with organizations play out. This fuels a realization that unless they act now, influencing the on-going interactions as they are made, they will continue to lose ground to organizations and any future efforts to lower their visibility will become futile. This leads to a variety of behaviors and actions that are purposefully enacted to better their situation and attempt to take control of the situation. They choose to adopt an active position in certain situations and become committed players who seek to exercise greater levels of agency by acting in ways that they anticipate will either limit organizations’ abilities to generate a meaningful assessment of their behaviors and preferences or provide sufficient benefits in exchange for that information they choose to share. This transition from awareness and understanding to commitment and game play is critical for the future of the game, as it drives the interactions forward and forces organizations to reflect on their existing digital structures, the ethicality of their digital business models and consumers’ rights to digital privacy, which are often enshrined in law. The game-like conditions are further accentuated by consumers’ mistrust in organizations’ intentions, as they feel suspicious, at times even cynical, about how organizations acquire and use their information for their own benefit (Martin, 2018).

The enactment of game playing behaviors does, however, not mean that these consumers feel fully able to manage when organizations ‘see’ them and how much of them is made visible. It was prevalent that although these consumers are making an active effort to use different concealment and cover moves, there is also a sense that this effort is not always sufficient to attain a dominant position in their online interactions with organizations. There were some signs of digital resignation (Draper and Turow, 2019) and a feeling that, in some situations, the effort put in to manage their digital traces was a somewhat lost cause as their actions might not change the power dynamic and produce the desired outcomes and levels of privacy (Altman, 1977) and many continue fatefully enacting at best a rearguard defense.

It is an acquired state to become an active participant in what we refer to as *Game Play* where consumers act on their *Game Awareness*, *Rule Familiarization*, and *Player Commitment* to either lower or heighten their online visibility depending on what the consumer believes they can gain from sharing their information with the organization; the level of trust the consumer has in the organization, and the amount of effort required to make oneself less visible. Importantly, the P2G framework also incorporates two feedback loops which illustrate the dynamic logic of the framework and the continuous work required by consumers to understand new methods and techniques organizations use. Some consumers re-engage with the Game Awareness and Rule Familiarization states to ‘top-up’ their understanding and knowledge of the evolving digital realities.

The digital consumer-organization touchpoints continue to be shaped by differing interests, technologies, aspirations, and intentions which form the game that consumers and organizations play. Consequently, consumers and organizations co-exist and engage in an ‘interactive dance’ (Goffman, 1969) as both parties make use of game-like calculations and tactical moves as they each seek to acquire, reveal, and conceal information. Critically, the research has surfaced what is at stake for consumers in their quotidian experience of inhabiting digital spaces and what they feel is worth fighting for in that space as it currently presents itself. In that sense it stands in somewhat stark contrast to the legal niceties and formalities of regulatory efforts to govern digital spaces, and information and privacy rights more generally, reflecting the dynamic and often opaque experience of the ever more ubiquitous digital dimensions of our lives.

## Conclusion and Implications

Digital spheres are contentious spaces where consumers’ and organizations’ interests do not always align. In many situations, this seeds mistrust and skepticism on the part of consumers toward organizations’ practices, intentions, and behaviors. Our investigation and its analysis offer a fruitful framework for thinking through the ways in which this state of affairs instantiates a dynamic between this consumer group and organizations in which an increasing propensity to game is all but inevitable. Given this set up, there is no simple advice available for organizations about how best to proceed. Whatever they do cannot but form another move in an ongoing game, just as our data are undoubtedly shaped by how our participants situated us when we sought their views on the practices they engaged in when orchestrating their digital interactions. They undoubtedly sought to tell us what they thought would be of interest to us, although in most cases they did appear to be thinking through their responses as they formulated them. Indeed, a number of them told us in the closing stages of our conversations that now they had engaged in the sorts of reflection our methods encouraged, they would likely further refine their online practices in the light of what had become more explicitly to the fore of their awareness as a result.

Our sample was purposively selected to ensure that it was made up of those who had likely been well socialized as digital natives. Other individuals, with other characteristics may articulate their experience otherwise in relation to the themes of our interest here. We by no means claim that all users of digitized spaces and modes of interaction with (partially) digitized organizations will proceed through the states of awareness and propensity for action that we have discerned in our sample group. Nor do we see the processes we describe as necessarily always accretive for the individuals involved in them. Context can and does matter, even in the most banal terms. A late night online shopping decision, possibly fuelled by preceding consumption of alcohol, may well take place without much awareness or gaming, even if at other times and in other places the purchaser concerned might enact many of the suite of moves that we delineate. At some times privacy and conscious control of self-visibility will be front of mind and at the heart of action. At others it won’t. But it does seem likely that higher states of playing the game, with more elaborate moves, will be less available to those who have not at some point progressed through the lower states of awareness that our P2G framework identifies. Future studies could explore how consumer groups with different characteristics to our participants engage with the four stages in the P2G framework while also looking to detect the potential influence of category data/information on consumers’ gaming activities.

Our talk of gaming also implicitly carries an anthropomorphism that is likely becoming more and more misplaced as circumstances develop. The immediate player that a consumer faces in many digitized interactions will increasingly be a model or algorithm carrying capacity for its own responsive decisions in the face of consumer moves, all the more so the more machine intelligence is built in. Perhaps, as this future develops, users will no longer articulate ponderous Data Subject Access Requests and the like in the realm of the right to know, only to wait for a doubtless legally compliant response that leaves them feeling that they are still really none the wiser about how, why and for what the information being kept on them is being gathered and used. Rather they may begin to fight fire with fire, unleashing intelligent algorithms of their own not only to occlude those parts of themselves that they wish to hold back but also to repeatedly request to know what is being done with the traces of themselves already captured. When there is no final whistle, there can be no final result. Once the game is afoot, the game is ongoing.

## Data Availability Statement

The original contributions presented in the study are included in the article/supplementary material, further inquiries can be directed to the corresponding author.

## Ethics Statement

This study was reviewed and approved by the Ethics Committee at Ashridge Executive Education at Hult International Business School. The patients/participants provided their written informed consent to participate in this study in accordance with the Declaration of Helsinki.

## Author Contributions

All authors listed have made a substantial, direct, and intellectual contribution to the work, and approved it for publication.

## Conflict of Interest

The authors declare that the research was conducted in the absence of any commercial or financial relationships that could be construed as a potential conflict of interest.

## Publisher’s Note

All claims expressed in this article are solely those of the authors and do not necessarily represent those of their affiliated organizations, or those of the publisher, the editors and the reviewers. Any product that may be evaluated in this article, or claim that may be made by its manufacturer, is not guaranteed or endorsed by the publisher.
